# Roux-en-Y Gastric Bypass Modulates AMPK, Autophagy and Inflammatory Response in Leukocytes of Obese Patients

**DOI:** 10.3390/biomedicines10020430

**Published:** 2022-02-12

**Authors:** Zaida Abad-Jiménez, Sandra López-Domènech, Celia García-Gargallo, Teresa Vezza, Segundo Ángel Gómez-Abril, Carlos Morillas, Pedro Díaz-Pozo, Rosa Falcón, Celia Bañuls, Víctor M. Víctor, Milagros Rocha

**Affiliations:** 1 Department of Endocrinology and Nutrition, University Hospital Doctor Peset, Foundation for the Promotion of Health and Biomedical Research in the Valencian Region (FISABIO), 46017 Valencia, Spain; zaiaji@alumni.uv.es (Z.A.-J.); cegarga3@alumni.uv.es (C.G.-G.); vezzateresa@gmail.com (T.V.); carlos.morillas@uv.es (C.M.); pedrodpas2@gmail.com (P.D.-P.); rosafalcon16@gmail.com (R.F.); celia.banuls@uv.es (C.B.); 2 Department of General and Digestive System Surgery, University Hospital Doctor Peset, Foundation for the Promotion of Health and Biomedical Research in the Valencian Region (FISABIO), 46017 Valencia, Spain; sean99cartu@yahoo.com; 3 Department of Surgery, Faculty of Medicine and Dentistry, University of Valencia, Av Blasco Ibáñez 13, 46010 Valencia, Spain; 4 CIBERehd-Department of Pharmacology, University of Valencia, Av Blasco Ibáñez 15, 46010 Valencia, Spain; 5 Department of Physiology, Faculty of Medicine and Dentistry, University of Valencia, Av Blasco Ibáñez 13, 46010 Valencia, Spain

**Keywords:** autophagy, inflammation, leukocytes, obesity, RYGB

## Abstract

Obesity is characterized by low-grade chronic inflammation, metabolic overload, and impaired endothelial and cardiovascular function. Roux-en-Y gastric bypass (RYGB) results in amelioration of the pro-oxidant status of leukocytes and the metabolic profile. Nevertheless, little is known about the precise mechanism that drives systemic and metabolic improvements following bariatric surgery. In this cohort study, we investigated the effect of RYGB on molecular pathways involving energy homeostasis in leukocytes in 43 obese subjects one year after surgery. In addition to clinical and biochemical parameters, we determined protein expression of systemic proinflammatory cytokines by Luminex^®^, different markers of inflammation, endoplasmic reticulum (ER) stress, autophagy/mitophagy by western blot, and mitochondrial membrane potential by fluorescence imaging. Bariatric surgery induced an improvement in metabolic outcomes that was accompanied by a systemic drop in hsCRP, IL6, and IL1β levels, and a slowing down of intracellular inflammatory pathways in leukocytes (NF-κB and MCP-1), an increase in AMPK content, a reduction of ER stress (ATF6 and CHOP), augmented autophagy/mitophagy markers (Beclin 1, ATG5, LC3-I, LC3-II, NBR1, and PINK1), and a decrease of mitochondrial membrane potential. These findings shed light on the specific molecular mechanisms by which RYGB facilitates metabolic improvements, highlighting the relevance of pathways involving energy homeostasis as key mediators of these outcomes. In addition, since leukocytes are particularly exposed to physiological changes, they could be used in routine clinical practice as a good sensor of the whole body’s responses.

## 1. Introduction

Obesity is a chronic low-grade inflammatory disease characterized by an imbalance between excessive intake and low expenditure of energy, which leads to metabolic overload. Obesity, per se, is associated with lower life expectancy, mainly due to related comorbidities, including metabolic (insulin resistance (IR), type 2 diabetes (T2D), and dyslipidemia) and cardiovascular disorders (hypertension, stroke, and endothelial dysfunction), musculoskeletal complications, physical disabilities and limitations, diverse mental illnesses, and cancer [1]. Strategies to treat obesity, including lifestyle interventions and pharmacotherapy, often produce unsatisfactory results [2]. In contrast, Roux-en-Y gastric bypass (RYGB) is a weight loss surgical technique that has been shown to bring clear health benefits to obese patients [3], including improvement of classic metabolic syndrome outcomes as well as inflammatory and subclinical atherosclerotic parameters [4,5,6]. However, the underlying molecular mechanisms mediating these clinical improvements are poorly understood.

Chronic low-grade inflammation is an intrinsic characteristic of obesity involving adipose tissue and leukocytes. Ectopic storage of fat stimulates the secretion of proinflammatory cytokines, such as tumor necrosis factor α (TNFα), interleukin 1β (IL1β), and interleukin 6 (IL6), which initiates an inflammatory cascade in leukocytes through Toll-like receptors (TLR) and nuclear factor kappa-light-chain-enhancer of activated B cells (NF-κB) signalling, resulting in the release of several chemokines, including monocyte chemoattractant protein-1 (MCP-1). In turn, MCP-1 promotes macrophage infiltration in metabolic tissues and prolongs and exacerbates the inflammatory response, thus helping to maintain IR [7].

Considering the metabolic role of AMP-activated protein kinase (AMPK) in insulin signalling and inflammatory pathways, it has also been proposed as a key pathogenic factor involved in obesity. Genetic ablation of the hematopoietic AMPK β1 subunit has been shown to increase adipose tissue macrophage infiltration and inflammatory markers and to reduce rates of fatty acid oxidation [8], while increased AMPK activity enhances lipid metabolism and anti-inflammatory actions in the liver and macrophages, offering protection against diet-induced obesity and the insulin resistant phenotype [9,10,11]. 

On the other hand, AMPK—one of the main metabolic sensors involved in energy homeostasis—enables cellular metabolism to adapt in response to nutritional challenges. In particular, AMPK is activated by physiological or pathological inputs involving ATP depletion, thereby inducing catabolic cellular processes [12]. Thus, under caloric restriction, AMPK activation promotes glucose uptake and glycolysis and activates lipolysis and oxidation. These changes are associated with a significant up-regulation of mitochondrial metabolism, mitophagy and autophagy and the attenuation of protein translation, resulting in the alleviation of endoplasmic reticulum (ER) stress [13,14].

In response to ER stress, the unfolded protein response (UPR) is activated through three different branches—inositol-requiring 1α (IRE1α), double-stranded RNA-dependent protein kinase (PKR)-like ER kinase (PERK) and activating transcription factor 6 (ATF6)—to prevent a rise in misfolded proteins and to activate the ER-associated degradation(ERAD) system and/or autophagy. However, persistent activation of UPR pathways during metabolic overload can lead to pathological events, including impairment of insulin signaling, initiation of inflammatory cascades [15], or even expression of CCAAT/enhancer-binding protein (C/EBP) homologous protein (CHOP), a transcription factor involved in the modulation of numerous pro-apoptotic factors [16,17]. In this context, it has been reported that ER stress is reduced in the adipose tissue of obese subjects after bariatric surgery, suggesting an association between ER stress relief and metabolic improvements in these patients [18,19].

For its part, autophagy is a catabolic process with an important role, not only in the recycling of cytosolic macromolecules and damaged cellular organelles, but also as an energy sensor for cell survival [20]. This process involves the formation and elongation of an isolation membrane driven by Phosphatidylinositol 3-kinase catalytic subunit type 3 (PI3KC3)/Phosphatidylinositol 3-kinase (VPS34)/Beclin 1 complex and different autophagy-related enzymes (ATGs proteins), catalysing the conjugation and lipidation of microtubule-associated protein 1A/1B-light chain 3 (LC3)-I to LC3-II, which acts as a bridge between the ubiquitinated cargo and autophagosome. Similarly, the selective engulfment of damaged mitochondria by autophagosomes—referred to as mitophagy—is driven principally by parkin RBR E3 ubiquitin ligase (PRKN) and serine/threonine kinase phosphatase and tensin homolog (PTEN)-induced putative kinase 1 (PINK1). When mitochondrial membrane potential is disrupted, PINK1 accumulates on the outer mitochondrial membrane and initiates a signalling cascade through mitochondrial clearance, thereby controlling mitochondrial quality and integrity [21]. Previous studies have described increased expression of autophagy markers in adipose tissue of obese subjects [22,23,24], that decreased after body mass reduction following bariatric surgery [23]. In contrast, reduced autophagic flux has been reported in isolated subcutaneous adipocytes from obese patients compared with non-obese controls, and weight loss after bariatric surgery was found to partially ameliorate autophagy of adipocytes [25]. However, studies of autophagy pathways in leukocytes are scarce [26,27] and, as far as we are aware, none of them has addressed this issue after weight loss induced by bariatric surgery.

Given the well-known role of AMPK as a metabolic sensor and its involvement in the inflammatory response and IR in obesity, it is possible that the improvement of these metabolic outcomes after RYGB is mediated by alterations in AMPK activity and downstream molecular pathways. Therefore, the aim of the present study was to explore whether RYGB-induced weight loss modulates AMPK activity and, following UPR, autophagy and mitophagy in leukocytes of obese patients.

## 2. Materials and Methods

### 2.1. Study Population

The present study was conducted in a sub-cohort of patients selected from a larger study of patients with obesity who underwent RYGB [6], registered in clinicaltrials.gov (accessed on 12 January 2022) under study number NCT05071391. All the study subjects were recruited between January 2017 and September 2019 from the Outpatients Clinic of the Endocrinology and Nutrition Service and the Department of General and Digestive System Surgery of the University Hospital Doctor Peset in Valencia (Spain). This prospective cohort study was carried out using 43 patients with a body mass index (BMI) ≥ 30 kg/m^2^ who were prescribed bariatric surgery to treat their obesity and related comorbidities. Patients aged 18 or older were eligible for inclusion. Exclusion criteria were pregnancy or lactation, severe renal or hepatic disease, history of drug abuse, previous history of cardiovascular or inflammatory diseases, and secondary obesity (hypothyroidism, Cushing’s syndrome). 

The study complied with the Declaration of Helsinki. The hospital’s Ethics Committee approved all the procedures involving patients (code 96/16; October, 2016). Written informed consent was obtained from all the participants. 

### 2.2. Clinical and Biochemical Determinations

All variables were determined at baseline and one year after the RYGB intervention. Systolic and diastolic blood pressure, weight, height, and waist circumference were recorded and BMI was calculated. Percentage of excess weight loss (EWL) was determined by the formula [(preoperative weight − current weight)**/**(preoperative weight − ideal weight (considering BMI = 25 kg/m^2^))] × 100.

Serum levels of glucose, total cholesterol (TC), high-density lipoprotein (HDL) cholesterol and triglycerides (TG) were measured by an enzymatic method, using a Beckman LX-20 analyser (Beckman Coulter, La Brea, CA, USA), and low-density lipoprotein (LDL) cholesterol was measured by Friedewald’s formula. Glycated haemoglobin (HbA1c) was measured with a glycohaemoglobin analyser (Arkray Inc., Kyoto, Japan). Insulin levels were determined by an immunochemiluminescent assay and IR status by the Homeostatic Model Assessment for IR index (HOMA-IR) formula ((fasting glucose in mg/dL × fasting insulin in μUI/mL)/405). Systemic levels of high sensitivity C-reactive protein (hsCRP) were analysed by means of an immunonephelometric assay (Behring Nephelometer II, Dade Behring, Inc., Newark, DE, USA) (intra-assay coefficient variation (CV) < 5.5%).

### 2.3. Evaluation of Systemic Cytokines IL6 and IL1β

Plasma samples were obtained by centrifuging (1500× *g*, 10 min, 4 °C) blood that was collected in EDTA-coated tubes and immediately stored at −80 °C. Levels of the proinflammatory cytokines IL6 and IL1β were then evaluated with a Luminex^®^ 200 analyser system (Luminex Corporation, Austin, TX, USA) according to the MILLIPLEX® Kit manufacturer’s procedure (Millipore Corporation, Billerica, MA, USA). All samples were evaluated twice, resulting in intra- and inter-serial CV of < 5.0% and < 15.0%, respectively.

### 2.4. Isolation of Leukocytes from Blood Samples

In order to isolate peripheral blood mononuclear cells (PBMCs) and polymorphonuclear leukocytes (PMNs), citrated blood samples were incubated with dextran 3% at room temperature (RT) for 45 min. The supernatant was then placed over Ficoll-Hypaque (GE Healthcare, Uppsala, Sweden) and then centrifuged for 25 min at 650× *g*. The resulting halo of PBMC was collected and centrifuged for 10 min at 650× *g*. The PMN pellet was incubated with Red Blood Cell Lysis Buffer (Sigma-Aldrich, Inc., St. Louis, MO, USA). PBMCs and PMNs were washed and resuspended in Hank’s Balanced Salt Solution (HBSS; Capricorn, Ebsdorfergrund, Germany) for subsequent analysis.

### 2.5. Fluorescence Imaging of Mitochondrial Membrane Potential

Mitochondrial membrane potential was determined by fluorescence static cytometry, using an IX81 Olympus microscope and ScanR version 2.03.2v software (Olympus, Hamburg, Germany). In short, PMNs were seeded in duplicate in 48-well plates and then incubated (30 min, 37 °C) with tetramethylrhodamine methyl ester (TMRM) and Hoechst 33,342 (Thermo Fisher Scientific, Waltham, MA, USA) for nuclei visualization. A total of 16 images per well were analysed.

### 2.6. Immunoblotting

Leukocytes were lysed for 15 min on ice with a buffer containing 20 mM HEPES pH 7.5, 0.4 M NaCl, 20% glycerol, 0.1 mM EDTA, 10 µM Na_2_MoO_4_, 0.5% NP-40, 1 mM dithiothreitol and a protease inhibitor mix constituted by 10 mM NaF, 1 mM NaVO_3_, 10 mM PNP and 10 mM β-glycerolphosphate. Next, samples were vortexed for 30 s and centrifuged at 16,100× *g* for 15 min at 4 °C. Total protein was then estimated by BCA assay (Thermo Fisher Scientific, Waltham, MA, USA). A total of 25 μg of protein was resolved in SDS-polyacrylamide gel by electrophoresis and then transferred onto a nitrocellulose membrane. After blocking the membranes for 1 h with 5% skimmed milk in TBS-T, the proteins of interest were detected by overnight blotting at 4ºC with the following antibodies: monoclonal anti-autophagy related protein 5 (ATG5) and polyclonal anti-LC3A/B (Cell Signaling Technology, Danvers, MA, USA), monoclonal anti-Beclin 1, monoclonal anti-AMPKα1α2, monoclonal anti-AMPKα1 (phospho T183) α2 (phospho T172), monoclonal anti-ATF6, and polyclonal anti-MCP-1 (Abcam, Cambridge, UK), monoclonal anti-CHOP and monoclonal anti-NF-κB (Thermo Fisher Scientific, Waltham, MA, USA), polyclonal anti-PINK1 (Sigma-Aldrich, Inc., St. Louis, MO, USA), and polyclonal anti-neighbour of BRCA1 gene 1 (NBR1) (Proteintech, Rosemont, IL, USA). Monoclonal anti-actin (Sigma-Aldrich, St. Louis, MO, USA) was incubated as a protein loading control. Secondary antibodies HRP-goat anti-rabbit (Millipore Iberica, Madrid, Spain) or HRP-goat anti-mouse (Thermo Fisher Scientific, Waltham, MA, USA) were then incubated for 1 h at RT and the chemiluminescence signal was detected by adding ECL Plus reagent (GE Healthcare, Little Chalfont, UK) or SuperSignal™ West Femto (Thermo Fisher Scientific, Waltham, MA, USA) to the membranes. Visualization was carried out using a Fusion FX5 Acquisition System, and the software Bio1D version 15.03a (Vilbert Lourmat, Marne-la-Vallée, France) was used to quantify the signal by a densitometry method.

### 2.7. Statistical Analysis

This study was designed to achieve a power of 80% and to detect significant (*p* < 0.05) differences of 20% in relation to the primary efficacy criterion—protein detection by western blot—assuming a common SD of 25 units. Accordingly, a minimum of 13 patients was required, as a loss to follow-up rate of 0% was estimated. For the statistical analysis of variables before and after the surgical intervention, we employed the statistics program SPSS 20.0 software (SPSS Statistics Inc., Chicago, IL, USA). Normal distribution was assessed by the Shapiro-Wilk test. In the case of parametric data, variables are represented as mean ± standard deviation (SD) (in table) or mean + standard error (SE) (in figures), and as median and 25th to 75th percentiles for non-parametric data; qualitative results are expressed as percentages. All data were analysed using a paired Student’s t-test (parametric data) or Wilcoxon test (non-parametric data) to compare variables at baseline and at one year follow-up. The confidence interval was 95% for all tests, and variables were considered significantly different when *p* < 0.05.

## 3. Results

### 3.1. Anthropometric and Biochemical Parameters following RYGB Surgery

This study was carried out in an obese cohort of 43 patients undergoing RYGB surgery (mean age 45.1 ± 11.4 years). Women constituted 84% of the total population (Table 1).

Patients undergone RYGB surgery showed a significant reduction in their BMI (*p* < 0.001), waist circumference (*p* < 0.001), systolic blood pressure (SBP), and diastolic blood pressure(DBP) (*p* < 0.01), which were accompanied by an improvement in glucose metabolism parameters—glucose, insulin, HOMA-IR, and HbA1c (*p* < 0.001 for all). Lipid profiles showed a significant decrease in TC content (*p* < 0.01), LDL cholesterol (*p* < 0.001), TG (*p* < 0.01), and a rise in HDL cholesterol levels (*p* < 0.001). When we focused on an acute phase inflammatiory reactant—hsCRP (*p* < 0.001)—and systemic cytokines—IL6 and IL1β (*p* < 0.05, for both)—we observed a significant decline in these inflammatory mediators.

As a whole, we observed a marked reduction in cardiovascular disease risk factors and systemic inflammatory parameters in obese patients one year after RYGB.

### 3.2. Inflammatory Response, AMPK Activation and ER Stress Markers in Leukocytes following RYGB Surgery

**Figure 1:**
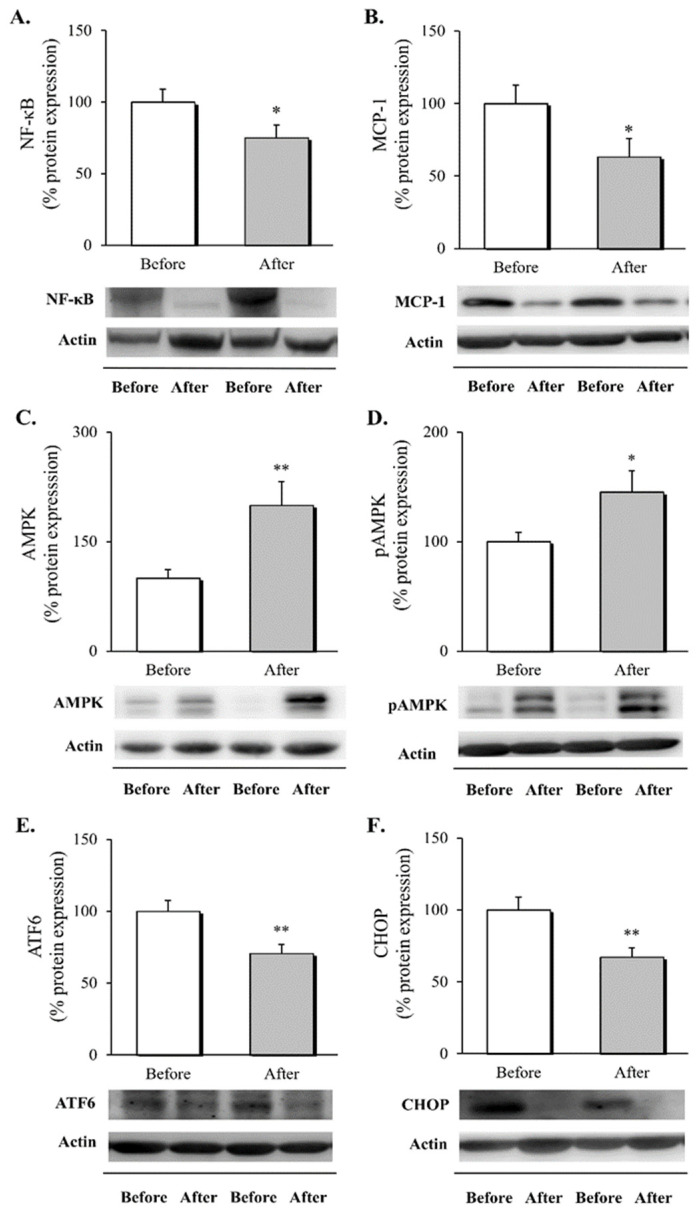
Evaluation of markers related to inflammation, AMPK expression, and ER stress in leukocytes of obese patients at one year follow-up. Protein expression and representative western blot images of (**A**) the transcription factor NF-κB (*n* = 22), (**B**) MCP-1 (*n* = 14), (**C**) AMPK (*n* = 18), (**D**) pAMPK (*n* = 18), ER components (**E**) ATF6 (*n* = 30) and (**F**) CHOP (*n* = 29). Data are expressed as mean + standard error, * *p* < 0.05 and ** *p* < 0.01 when compared using a two-sided paired Student’s t-test. (p)AMPK, (phosphorylated)AMP-activated protein kinase; ATF6, activating transcription factor 6; CHOP, CCAAT/enhancer-binding protein (C/EBP) homologous protein; ER, endoplasmic reticulum; NF-κB, nuclear factor kappa-light-chain-enhancer of activated B cells; MCP-1, monocyte chemoattractant protein 1.

To determine the impact of RYGB on intracellular inflammatory pathways, activation of AMPK and ER stress markers, we evaluated different protein mediators in leukocytes before and after surgery (Figure 1).

The results show a decrease in NF-κB, which plays a central role in inflammation through its ability to induce transcription of proinflammatory genes, including MCP-1 (Figure 1A,B, *p* < 0.05 for both), one year after surgery. The effect of the surgical procedure on the activation of AMPK was assessed in leukocytes by quantifying total AMPK protein expression and its activation through T183 and T172 phosphorylation. We observed a significant rise in total and activated AMPK (Figure 1C,D, *p* < 0.01 and *p* < 0.05, respectively), one year after the intervention. Finally, given the link between AMPK and the attenuation of protein translation, we evaluated changes in ATF6 and CHOP, markers involved in the UPR and apoptosis in response to chronic cellular stress, observing a drop in the protein expression levels of these mediators (Figure 1E,F, *p* < 0.01 for both). 

Overall, these results suggest that RYGB leads to an improvement in the inflammatory response, manifested by a down-regulation of leukocyte activation and chronic stress, that would seem to be mediated by AMPK activation.

### 3.3. Autophagy and Mitophagy in Leukocytes following RYGB Surgery

**Figure 2:**
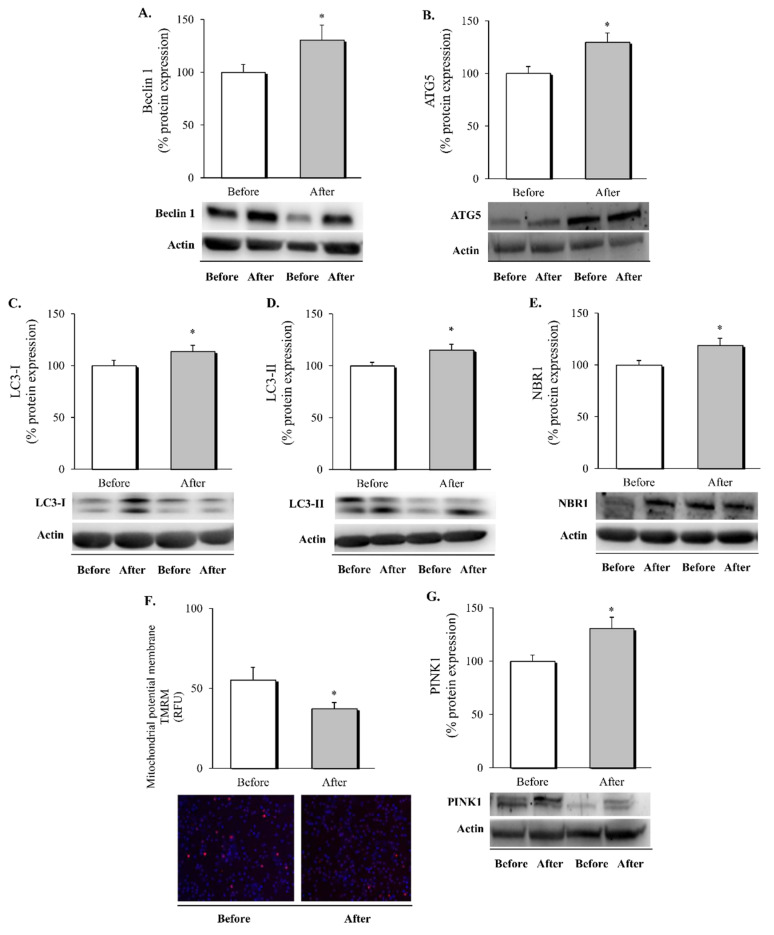
Autophagy and mitophagy markers evaluated in leukocytes of obese patients at one year follow-up. Protein expression and representative western blot images of (**A**) Beclin 1 (*n* = 21), (**B**) ATG5 (*n* = 21), (**C**) LC3-I (*n* = 19), (**D**) LC3-II (*n* = 19), (**E**) NBR1 (*n* = 16) and mitophagy marker (**G**) PINK1 (*n* = 20). Fluorescence microscopy images (100×) of (**F**) TMRM (*n* = 26) as an indicator of mitochondrial membrane potential. Data are expressed as mean + standard error, * *p* < 0.05 when compared using a two-sided paired Student’s t-test. ATG5, autophagy related 5; LC3, microtubule-associated protein light chain 3; NBR1, neighbour of Brca1; RFU, relative fluorescence units; TMRM, tetramethylrhodamine methyl ester; PINK1, serine/threonine kinase phosphatase and tensin homolog (PTEN)-induced putative kinase 1.

After analysing the impact of RYGB surgery on the inflammatory status and UPR of leukocytes, the next step was to specifically evaluate autophagy and mitophagy pathways in these cells (Figure 2).

We observed a significant increase in the expression of all the autophagy markers evaluated—Beclin 1, ATG5, LC3-I, LC3-II, and NBR1, including autophagy nucleation, elongation and, maturation of autophagosome (Figure 2A–E, respectively, *p* < 0.05 in all cases). In addition to the up-regulation of autophagy, we also sought to evaluate potential changes in mitophagy. Alteration of the mitochondrial membrane potential is considered the first signal of mitochondrion clearance. In this respect, we detected a decrease in the TMRM fluorescence signal (Figure 2F, *p* < 0.05) that was accompanied by an increase in PINK1 (Figure 2G, *p* < 0.05), suggesting a restoration of mitochondrial homeostasis following the surgical intervention.

These results provide evidence that RYGB induces intracellular clearance through the regulation of autophagy in leukocytes, improving mitochondrial turnover as a consequence.

## 4. Discussion

In the present study we demonstrated that, in our cohort of middle-aged obese subjects, RYGB surgery promoted pronounced weight loss, accompanied by an improvement in metabolic and inflammatory parameters one year later. We observed an up-regulation of AMPK in circulating leukocytes after the intervention, and this was accompanied by an amelioration of proinflammatory signalling, ER stress and proapoptotic markers and the activation of cell clearance mechanisms through autophagy/mitophagy. Considering the essential role of AMPK as a metabolic regulator in obesity, our results suggest that RYGB stimulates adaptive responses in leukocytes that may facilitate the partial recovery of cellular homeostasis, with potential systemic effects on inflammation and IR. In sum, the present results represent novel and valuable evidence about the molecular mechanisms that seem to underlie the protective effects of bariatric surgery against metabolic comorbidities in obesity.

There is accumulating evidence that RYGB produces an improvement in lipid profile, loss of excess BMI and a lower risk of cardiovascular events, all of which are maintained one year after surgery [28] and remain for six [29] to eight years [30], thus having a durable impact on cardiovascular health. Clinical and metabolic parameters had improved considerably in our study population one year after surgery, with relevant decreases observed in blood pressure and systemic inflammation, and improvements in glucose homeostasis and lipid profile, all of which is in the line with the findings of previous studies. 

Moreover, weight loss induced by surgical intervention may modify regulators that control catabolic and anabolic cellular processes. We used leukocytes to evaluate these cellular pathways because they are particularly exposed to physiological changes and act as a sensor of the whole body’s responses to disease [31]. Specifically, it has been reported that the mitochondrial physiology of lymphocytes in response to the nutritional state is related to that of other cell types [32]. In addition, dysregulation of the immune cell response is a major contributor to chronic systemic inflammation in obesity, with deleterious consequences for insulin sensitivity and endothelial function [33].

Growing evidence has highlighted an important role for AMPK in the pathophysiology of obesity, particularly in the organs involved in energy metabolism, such as white adipose tissue, skeletal muscle and liver [9,34]. Focusing on adipose tissue, Luo et al. [35] observed that metformin-induced activation of AMPK reversed fat fibrosis and IR in obese mice, while suppression of AMPK contributed to a persistent aberrant extracellular matrix in human visceral adipose tissue. In obese patients, decreased AMPK activity has been associated with increased expression of inflammatory genes in adipose tissue and systemic IR [36], whereas weight loss intervention (caloric restriction) has been found to induce increased metabolic capacity and enhanced AMPK activation [37]. However, only a few studies have evaluated the effect of bariatric surgery on AMPK in humans. Increased expression and activity of AMPK was reported in subcutaneous adipose tissue 3–6 months after RYGB, and was accompanied by a reduction in oxidative stress, ER stress and inflammatory markers [19,38]. Similarly, AMPK activity was found to be increased in PBMCs one year after bariatric surgery—via laparoscopic bypass or sleeve gastrectomy—inducing chaperone-mediated autophagy, a reduction in protein oxidative damage and hsCRP [39,40]. In accordance with these results, our study highlights an increase in AMPK activity associated with a reduction in systemic inflammatory markers—IL6, IL1β, and hsCRP—and intracellular proinflammatory mediators—NF-κB and MCP-1—in leukocytes. Interestingly, AMPK inhibits the acute proinflammatory response by targeting the NF-κB signalling pathway in leukocytes [41], and AMPK activators relieve ER stress and MCP-1 in human monocytes/macrophages treated with palmitate or lipopolysaccharide (LPS) [42,43], which is also in line with our results. On the basis of all these findings, strategies that activate AMPK should be harnessed for ameliorating inflammatory pathways.

Pathological conditions involving inflammation have been implicated in the activation of ER stress. In this context, previous research has associated obesity with an increase in ER stress markers in subcutaneous adipose tissue that correlates with adiposity [44,45]. Moreover, Gregor et al. [18] reported reduced expression of ER stress markers in the liver (glucose-regulated protein 78 kDa (GRP78), phosphorylated eukaryotic translation initiation factor 2A (p-EIF2α)), subcutaneous adipose tissue (X-box binding protein 1 (sXBP1), GRP78, CHOP and p-EIF2α) one year after RYGB intervention. In line with this, Ferraz Bannitz et al. [19] observed a drop in ER stress expression (PERK and calreticulin(CALR)) in subcutaneous adipose tissue 3–6 months after surgery. Both studies described changes mainly in transcripts in subcutaneous adipose tissue; as far as we are aware, we are the first group to describe the effects of RYGB on ER stress protein markers in leukocytes. Our results are not only in accordance with those of the aforementioned reports but are also in line with our previous study describing the amelioration of chronic ER stress in leukocytes of obese patients after diet-induced weight loss, revealed by a diminished ATF6 and CHOP expression [46]. As a whole, these data demonstrate a significant regulation of ER stress in the weight loss provided by RYGB, and a possible link with amelioration of the inflammatory response [15].

Another important role of the AMPK pathway is the regulation of different phases of autophagy. On the one hand, AMPK reduces mechanistic target of rapamycin (mTOR) activity under conditions of energy stress [47]. Alternatively, AMPK may directly phosphorylate unc-51 like autophagy activating kinase 1 (ULK1) which is important, not only for autophagy, but also for the selective removal of damaged mitochondria [48], ATG9 [49], VPS34 and Beclin 1 [50]. However, few studies have evaluated the possible interaction between autophagy and weight loss in obese models. Previous research has shown that the downregulation of hepatic autophagy in high-fat diet-induced obesity promotes ER stress and IR [51,52], whereas RYGB is reported to improve hepatic lipid metabolism through an increase in the LC3-II/LC3-I ratio and the down-regulation of mTOR [53]. In the case of 3T3-L1 preadipocytes, the recent report published by Zhang et al. [54] points to autophagy as the molecular effector that restores lipid accumulation in adipocytes through activation of autophagy and chaperone-mediated autophagy. In accordance with these previous studies, we have detected an up-regulation of the autophagy markers involved in the nucleation, elongation, and formation of autophagosomes and cargo receptors—Beclin 1, ATG5-ATG12 complex, LC3, and NBR1, respectively—in leukocytes after RYGB. Altogether, these findings implicate the activation of autophagic pathways in the amelioration of obesity-associated comorbidities in metabolic tissues, whereas, in leukocytes, autophagy has an immunological function involving the control of inflammation (see [55] for review). In line with this, previous studies have shown that B cells present a skewed profile and lose the function by which they facilitate T cell inflammation after RYGB, which results in a significantly elevated frequency of anti-inflammatory IL10-producing cells and a reduced frequency of IL6-producing cells [56], in line with the systemic reduction of IL6 we report herein.

Mitophagy is not only regulated by AMPK through ULK phosphorylation [57], but also by PINK1/PRKN -mediated removal of depolarized mitochondria [58]. In the present study, we have shown that an increase in AMPK expression after RYGB is associated with a reduction in mitochondrial membrane potential and an increase in the mitophagy mediator PINK1. Furthermore, we have previously demonstrated in a larger cohort of obese patients that leukocytes undergo a drop in superoxide levels after RYGB [6], thus suggesting that mitochondrial clearance through mitophagy improves mitochondrial function after the intervention. In accordance with our findings, Kirwan and colleagues reported increased expression of the mitochondrial fusion protein mitofusin 1(MFN1) and the mitophagy marker Bcl-2/adenovirus E1B 19-kDa interacting protein (BNIP3) in the liver of obese rats following RYGB [59].

The principal strength of our study is that we have evaluated the molecular pathways that involve inflammation, autophagy, and stress sensors in the leukocytes of obese patients one year after RYGB. In addition, as far as we know, this is the first study that has evaluated these markers in human obese patients, and our results suggest an interconnection between AMPK activation, inflammation, autophagy, mitochondrial function, and ER markers. However, since causality cannot be inferred from our data, further analyses are required to determine how these intracellular signalling pathways are interrelated. Furthermore, our findings need to be corroborated over a long-term period. Finally, further prospective research employing direct and functional methods (e.g., confocal and electron microscopy) to assess autophagy flux and mitochondrial dynamics would help to reinforce the validity of our findings.

## 5. Conclusions

In summary, the results of the present study extend our understanding of the molecular mechanisms underlying the metabolic improvements that obese patients display when weight loss is achieved by RYGB. Interestingly, we show that the improvements in systemic inflammation and metabolic outcomes are mirrored by an increase in AMPK content, the attenuation of inflammatory markers and chronic ER stress, and the activation of cell clearance mechanisms through autophagy/mitophagy. Our findings highlight the relevance of restoration of leukocyte homeostasis—including different cellular signaling pathways—as a potential target in the treatment of metabolic complications of obesity. Therefore, since AMPK plays a central role in energy homeostasis, future research should be focused on development of strategies that activate this molecular pathway.

## Figures and Tables

**Table 1:** Anthropometric parameters before and after RYGB.

Parameters	Before	After
*n* (females %)	43 (83.7)	
Age (years)	45.1 ± 11.4	
Weight (kg)	108.7 ± 15.6	79.2 ± 13.0 ***
BMI (kg/m^2^)	39.6 ± 4.9	29.2 ± 4.4 ***
Waist (cm)	115.0 ± 10.2	88.6 ± 11.5 ***
EWL (%)		79.1 ± 30.6
SBP (mmHg)	133.2 ± 15.6	123.8 ± 17.3 **
DBP (mmHg)	81.4 ± 10.7	73.9 ± 9.8 **
Glucose (mg/dL)	98.7 ± 26.3	86.0 ± 12.3 ***
Insulin (μU/mL)	14.6 ± 7.8	7.1 ± 3.2 ***
HOMA-IR	3.8 ± 3.5	1.5 ± 0.7 ***
HbA1c (%)	5.5 ± 0.7	5.2 ± 0.5 ***
TC (mg/dL)	187.0 ± 33.6	166.9 ± 27.7 **
HDLc (mg/dL)	47.0 ± 8.9	55.1 ± 10.0 ***
LDLc (mg/dL)	122.6 ± 41.4	96.4 ± 21.4 ***
TG (mg/dL)	96 (74, 143)	78 (55, 100) **
hsCRP (mg/L)	3.7 (1.7, 6.3)	0.6 (0.3, 1.2) ***
IL6 (pg/mL)	4.0 ± 3.0	3.3 ± 2.3 *
IL1β (pg/mL)	1.2 ± 0.9	1.0 ± 0.7 *
**Treatment**		
Hypertension % (*n*)	34.8 (15)	9.3 (4)
Hyperlipidemia % (*n*)	23.3 (10)	9.3 (4)
T2D % (*n*)	30.2 (13)	4.6 (2)

Data are expressed as mean ± SD or percentage (*n*). TG and hsCRP are represented as median and IQ range (25th and 75th percentile). Values were statistically compared using a paired Student’s t-test or Wilcoxon test and were considered significant when * *p* < 0.05, ** *p* < 0.01, and *** *p* < 0.001. BMI, body mass index; DBP, diastolic blood pressure; EWL, excess weight loss; HbA1c, glycated haemoglobin; HDLc, HDL cholesterol; HOMA-IR, Homeostatic Model Assessment for IR index; hsCRP, high-sensitivity C-reactive protein; IL1β, interleukin 1β; IL6, interleukin 6; LDLc, LDL cholesterol; RYGB, Roux-en-Y gastric bypass; SBP, systolic blood pressure; TC, total cholesterol; TG, triglycerides; T2D, type 2 diabetes.

## Data Availability

The data presented in this study are available on request from the corresponding authors. The data are not publicly available due to ethical reasons.
